# Local Mechanical Properties and Microstructure of EN AW 6082 Aluminium Alloy Processed via ECAP–Conform Technique

**DOI:** 10.3390/ma13112572

**Published:** 2020-06-05

**Authors:** Radek Procházka, Peter Sláma, Jaromír Dlouhý, Pavel Konopík, Zuzanka Trojanová

**Affiliations:** 1 COMTES FHT, Průmyslová 995, 33441 Dobřany, Czech Republic; pslama@comtesfht.cz (P.S.); jdlouhy@comtesfht.cz (J.D.); pkonopik@comtesfht.cz (P.K.); ztrojan@met.mff.cuni.cz (Z.T.); 2 Faculty of Mathematics and Physics, Charles University, Ke Karlovu 3, 12116 Praha 2, Czech Republic

**Keywords:** ECAP–Conform, EBSD analysis, local mechanical properties, micro-tensile techniques

## Abstract

An ultrafine-grained EN AW 6082 aluminum alloy was prepared by continuous serve plastic deformation (i.e., thermo-mechanical equal channel angular pressing (ECAP)–Conform process). A miniaturized tensile testing technique was used for estimating local mechanical properties with the aim to reveal the inhomogeneity of elastic and plastic properties in a workpiece volume. These inhomogeneities may appear due to the irregular shear strain distribution in a Conformed wire. Miniaturized samples for tensile testing were cut from the Conformed workpiece. Elongation of miniaturized samples was measured with a 2D digital image correlation technique as the optical extensometer. Tensile test characteristics, such as the yield strength and ultimate tensile strength, were consequently compared with results of conventional and hardness tests. The microstructure of Conformed bars was studied in the cross-section perpendicular and parallel to the extrusion direction using scanning electron microscope (SEM) and electron backscatter diffraction (EBSD) analysis. The microstructure of samples exhibits pronounced inhomogeneity, which is reflected by the hardness and tensile test results. Estimated distinctions between peripheral and central parts of the Conformed wires are probably a consequence of the significant strain differences realized in the upper and bottom wire parts.

## 1. Introduction

In the last decades, numerous methods of severe plastic deformation (SPD) have been developed for achieving significant strengthening due to grain refinement [1,2,3,4,5]. The application of SPD procedures to commercial aluminum alloys showed that each alloy was strengthened after SPD processing by factor of two or three [6]. Among different SPD methods, the equal channel angular pressing (ECAP)–Conform method provides a possibility for the continuous production of bulk ultrafine-grained (UFG) materials [7,8]. Resulting material properties depend, besides on the tool geometry, also on the procedure parameters (processing temperature, strain rate, back stress) [9,10,11,12]. This is reason why, beside experiments, significant effort is devoted to modeling of SPD processes and computational simulations [13,14]. Considerable improvement of material properties is usually recorded even after the first ECAP–Conform pass [8,15,16]. On the other hand, inhomogeneity and anisotropy of the processed materials is often observed [17,18]. Conventional mechanical testing of such inhomogeneous samples might produce misleading results and significant scatter of experimental data. Miniature specimen test techniques are suitable for materials prepared with SPD methods where only a small amount of the material is processed, such as high-pressure torsion (HPT), accumulative roll bonding (ARB), and equal channel angular pressing (ECAP) [19]. On the other hand, this technique can be also used in case of long and thin products (ECAP–Conform and Conform) where processed materials can be tested only in one direction using a conventional technique. Due to the small ingot sizes, the investigation of material behavior cannot be done with current testing techniques. The investigation of mechanical properties in ultrafine or nano-grained materials is in many cases restricted to an indirect evaluation, such as a combination of the (micro) hardness measurement with microstructure evaluations [7]. Unfortunately, this approach is unable to describe elastic or plastic behavior during the loading, which provides the experimental data necessary for computer modeling. A small amount of the experimental material and local mechanical properties can be therefore investigated by miniature flat tensile specimens [19,20,21]. This method may be applied for micro- or ultrafine-grained samples where a large number of grains per sample cross-section ensures good reproducibility of experimental results. The axial tensile stress–strain (S-S) characterization of SPD materials can be obtained by using a precise load cell of the testing machine and a digital image correlation (DIC) method that provides sufficient accuracy for the material deformation during specimen loading [22,23,24,25,26].

The present investigation aims at critical evaluation of the ECAP–Conform process with special interest in the possible anisotropy of mechanical properties and microstructure inhomogeneities in an EN AW Al6062 alloy. Use of miniaturized testing techniques allowed us to prepare a set of flat tensile test specimens, with the stress axis oriented into three workpiece directions.

## 2. Material and Methods

The EN AW 6082 T6 (AlSi1MgMn) aluminum alloy, in the form of extruded wires with a diameter of 12 mm, was used in this study. This material is hereafter depicted as the initial state (IS). The chemical composition of the alloy, determined by a Bruker Q4 Tasman optical emission spectrometer (BRUKER AXS Inc., Madison, WI, USA), is given in Table 1.

**Figure 1:**
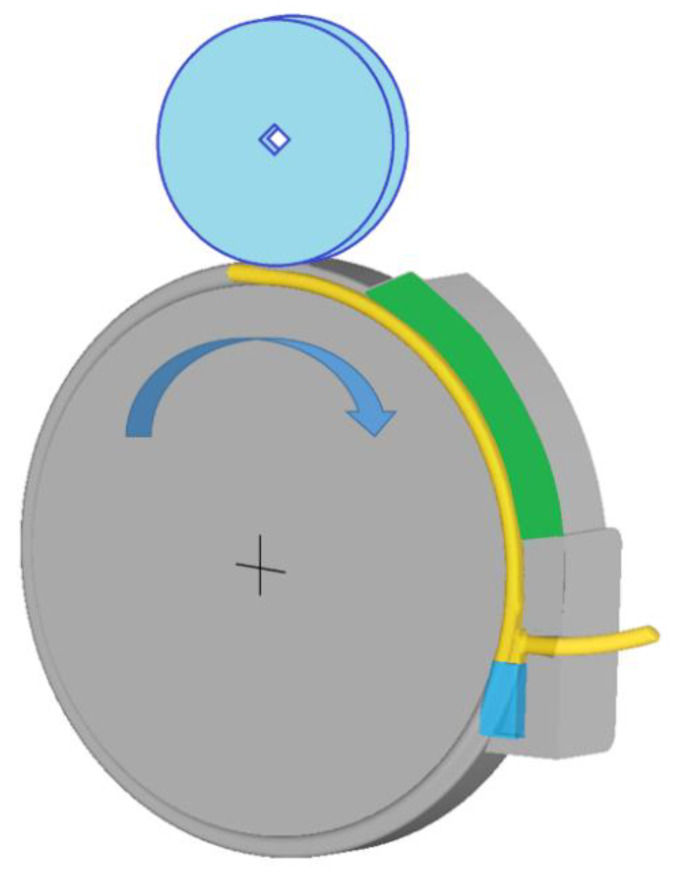
Schematic drawn of the ECAP–Conform process.

**Figure 2:**
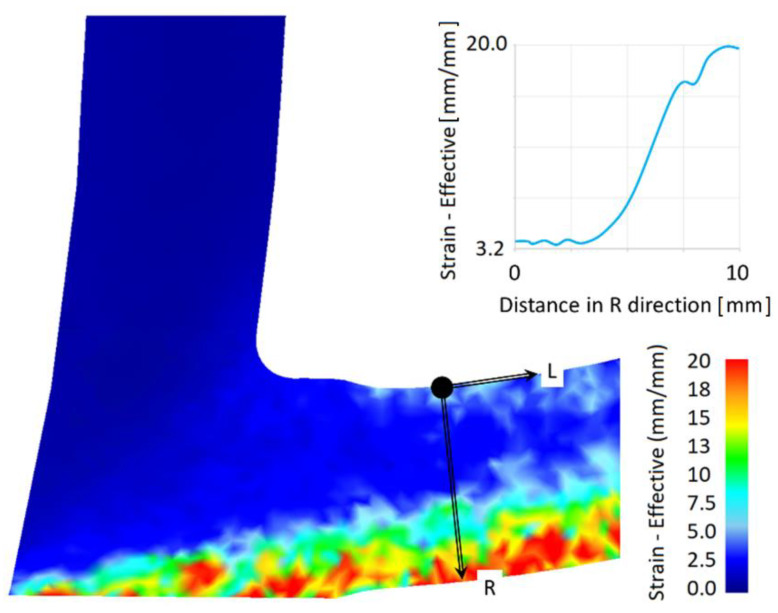
Numerical modelling of the ECAP–Conform process. Strain—Effective distribution after abutment and after diameter reduction of 31%.

The IS material was submitted to thermal treatment (HT) consisting of heating up to 530 °C with a holding time of 30 min and quenching into water of ambient temperature. Applying such a HT, the softening of the material was done and the quenched state (QS) was achieved. QS material was used as a feedstock for the presented ECAP–Conform processing. The QS material was put into the ECAP–Conform tool and processed at room temperature. The schematic drawn of the ECAP–Conform device is shown in Figure 1. The material enters into the facility with a speed of 15 m/s and is carried forward with frictional forces. An important point of the facility is the tool exit, where an abutment turns the rod through an angle ϕ = 90°. If strain is due to one pass through the ECAP tool with the angle 90°, ε ~ 1, it is reasonable to anticipate that the strain introduced in the ECAP–Conform tool will be approximately the same when the cross-sectional area is not changed [7]. The numerical modelling of the process is illustrated in Figure 2. The picture shows the strain distribution in the abutment, where the minimum effective strain of 3.2 mm/mm and the maximum effective strain of 20 mm/mm were reached close to the bottom surface, which corresponds to a distance 10 mm in the radial (R) direction. In this case, the rod diameter was reduced after one pass through the tool by 31%, and then it is necessary to calculate the additional strain for this extrusion process in accordance with Figure 2.

The efficiency of this single ECAP–Conform process depends on requirements associated with material losses, stability and homogeneity of the original microstructure. These requirements can be evaluated by means of microstructure analysis and micro-hardness tests.

The microstructure of samples in the QS and ECAP–Conformed state (ECS) was studied using the light microscope Zeiss Axio Observer Z1 (Carl Zeiss Jena GmbH, Jena, Germany).

The QS and Conformed material were further studied in the scanning electron microscope (SEM) Jeol JSM-IT500HR (JEOL Inc., Peabody, MA, USA) with a Hikari Super EBSD camera (EDAX Inc., Mahwah, NJ, USA), equipped for EDAX’s electron backscatter diffraction (EBSD) analysis. The orientation imaging microscopy (OIM) technique was used for imaging different crystallographic orientations of grains. The observations were performed on the perpendicular section to the extrusion direction (L). These investigations were realized after a standard metallographic preparation including polishing and electro-etching for observation in polarized light with Barker’s reagent (2% HBF_4_), using 20 V direct current for two min.

Micro-hardness evaluations of all three material states were performed in a micro-hardness tester Durascan-50 (Struers Inc., Cleveland, OH, USA) under the applied load of 0.5 kg (HV05). The dwell time exhibited was 20 s. The micro-hardness distribution over the entire sample cross-section makes visible possible distribution of mechanical properties and their scatter. Changes in the mechanical properties in the cross-sectional area can be visualized as a hardness profile and may verify results obtained with miniature flat tensile tests.

As a verification of suggested miniaturized tensile testing technique, standard tensile (STT) and micro-tensile tests (MTT) were carried out. Samples were cut from the three different material states (IS, QS and ECS). The sample orientation for the STT was given by the workpiece dimensions before (12 mm in diameter) and after ECAP–Conform processing (10 mm in diameter). All tensile tests were conducted at room temperature.

Standard tensile tests were realized on round test bars with the reduced gage section exhibiting 6 mm diameter and 35 mm of active length cut in the in the extrusion direction (L). The sample design was chosen according to ČSN EN ISO 6892-1. Central parts of bars were used for the test specimen manufacturing. Tensile tests were conducted in a servo-hydraulic testing system Inova with a load capacity of 200 kN. Samples were strained at a constant strain rate of 0.0004 s^−1^. The tests were performed according to the abovementioned standard. Elongation of deformed samples was measured using a mechanical extensometer having the gauge length of 25 mm. From the force extension diagrams and initial sample dimensions, engineering stress–strain (S-S) curves were constructed and characteristic stresses were obtained; the proof stress (YS), ultimate tensile strength (UTS) and Young modulus (E) were estimated.

**Figure 3:**
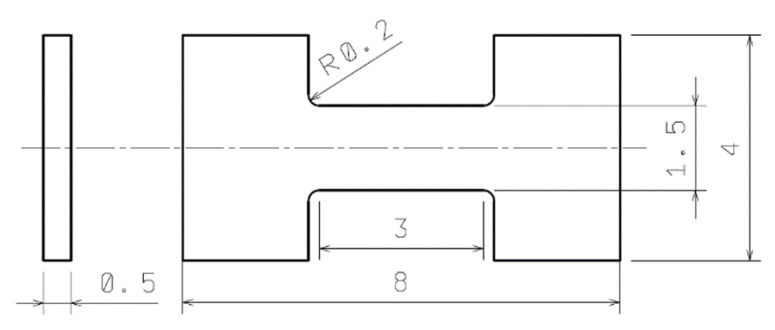
Samples geometry for MTT (mm).

Samples for miniaturized tests were carefully machined from wires to reach their final shape and dimensions (active length of 3.0 mm, width of 1.5 mm and thickness of 0.5 mm)—see Figure 3. The sample geometry is very similar to the one that was used for the experimental investigation of a magnesium alloy by Máthis [21], where the shorter active length was used. As the final step of specimen preparation, the surface roughness was polished. Subsequently, a pattern of stochastic speckles to the surface was deposited providing sufficient grayscale contrast for digital image correlation (DIC) measurements.

The geometry of miniature flat tensile samples was designed to fit into the available tangential dimensions of the wire, 10 mm in diameter. Their size is almost identical to the sample volume recommended for the small punch test [24,26]. Micro-tensile tests were performed at the same testing conditions as in the case of STT. A testing machine with a loading capacity of 10 kN was employed. The tensile load was applied to a sample until a 90% drop in the force occurred. The non-contact strain measurement using the DIC system is realized with a displacement accuracy about 0.2 μm for a 10 × 8 mm^2^ field of view.

Prior to testing, verification of the alignment of the testing machine was performed using alignment fixtures. This procedure eliminates the imposition of the samples’ bending strains and stresses under loading. Mechanical properties such as E modulus and characteristic stresses—YS and UTS—were directly obtained.

**Figure 4:**
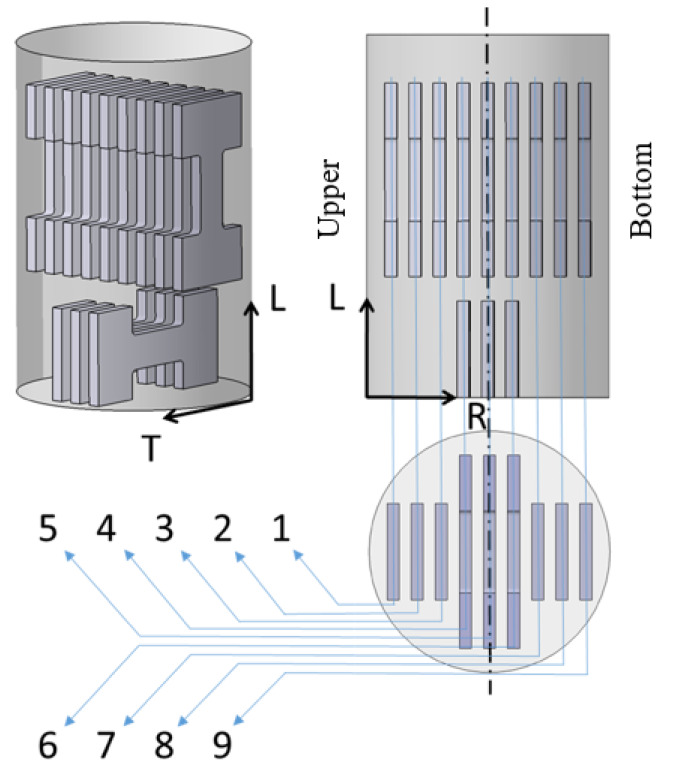
Cutting scheme of the processed wire.

The cutting scheme of processed wire with a diameter of 10 mm is depicted in Figure 4, where MTT specimens were cut out in the longitudinal direction (L), i.e., extrusion and the tangential (T) directions. The first (L1) and the last (L9) samples in the L direction were cut out of the wire workpiece close to its surface. The numbers presented in Figure 4 correspond to the distance in millimeters of the initial TLR coordinate axis system in the radial (R) direction which is related to the upper surface (U) of the wire. The specimen L5 represents the center of the wire. The same procedure was applied for sample cutting in the T direction.

For a specimen design of MTT, the effect of size and its proportionality played a less important role in determining tensile properties until the UTS point. This effect definitely causes a reduction in ductility, such as total elongation, and thus it might lead to undesirable material behavior. On the other hand, it does not affect the yield strength YS, ultimate strength UTS or Young’s modulus E.

## 3. Results

### 3.1. Microstructure of Samples

In case of the IS material microstructure, the results have been shown and published by Malecek [27] where the microstructure and texture distribution of extruded rods were investigated.

**Figure 5:**
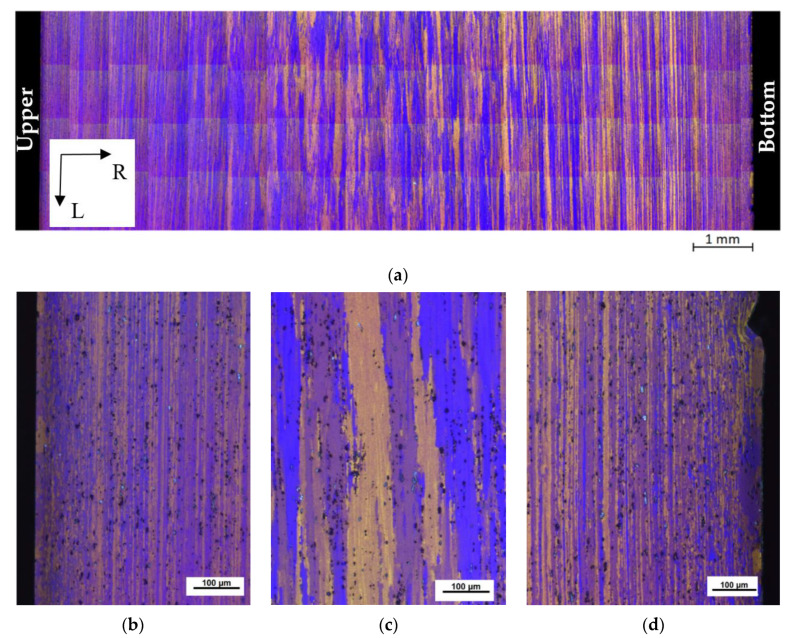
Light micrographs of the QS material taken on the section corresponding to the LR plane: (**a**) overall image of the microstructure; (**b**) first and (**d**) second surface sections showing long, thin grains near the surface; (**c**) massive grains in the wire center (Barker’s etching was used).

As a feed stock, the QS material was used for the ECAP–Conform treatment. The microstructure of the QS material is shown in Figure 5a. It can be seen that the wire microstructure is fibrous, with thin grains elongated in the extrusion direction near the wire surface; see Figure 5b,d. Massive grains (see Figure 5c) are situated in the wire center even if the HT was processed. No substantial differences in micrographs, taken in the vicinity of both surfaces, were observed, as is documented in Figure 5b,d. This microstructure corresponds to the microstructure of extruded materials generally or the IS material used in this study, respectively [27].

**Figure 6:**
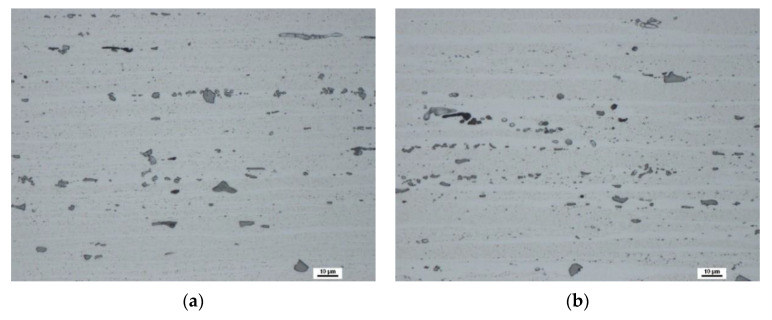
Electron micrographs of the QS material taken in the LR section: (**a**) surface region; (**b**) central region. Dix–Keller etching was used for revealing precipitates.

Scanning electron micrographs of the QS sample, taken on the LR section, are shown in Figure 6a (surface region) and Figure 6b (central region). The QS material exhibits a fibrous microstructure with grains elongated in the L direction. In the surface layer, the grains are more elongated and have a smaller thickness compared with the grains situated in the central region; see Figure 6. Coarse α-Al_15_(Fe,Mn)_3_Si_2_ and Mg_2_Si phases are arranged in rows parallel to the extruded direction (L). Besides coarse particles, small precipitates are visible in Figure 6. Precipitates free zones were found in the wire central part; see Figure 6b.

**Figure 7:**
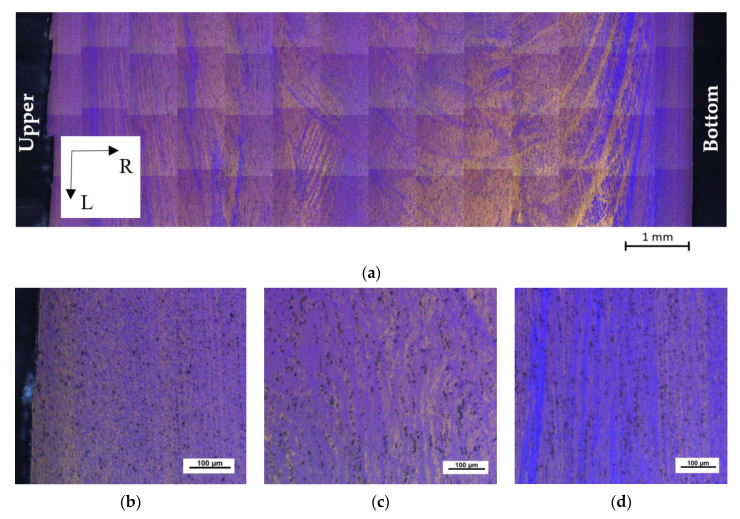
Material microstructure after ECAP–Conform processing taken on the LR section: (**a**) overall image; (**b**) upper and (**d**) bottom surface showing long, thin grains in the surface vicinity; (**c**) massive grains in the central region. Barker’s etching was used.

Figure 7 shows the microstructure of the ECS material in the LR section. While the bottom (B) surface of the workpiece seems to be smooth, the upper (U) surface exhibits higher roughness with several cracks. Long, thin grains elongated in the extrusion direction (L) are visible near the workpiece surface, Figure 7b,d, while the massive grains were found in the center, Figure 7c.

**Figure 8:**
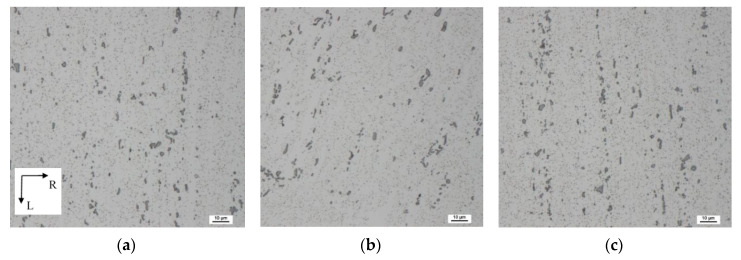
Electron micrographs of the ECS material taken on LR sections: (**a**) left surface region; (**b**) central region; (**c**) right surface region. Dix–Keller etching was used.

Figure 8 shows scanning electron micrographs of ECS material taken from the surface and central region. Coarse α-Al_15_(Fe,Mn)_3_Si_2_ and small Mg_2_Si precipitates are ordered in rows oriented to the L direction, especially in the peripheral parts of the processed wire. This indicates higher strains which are present in surface wire layers.

**Figure 9:**
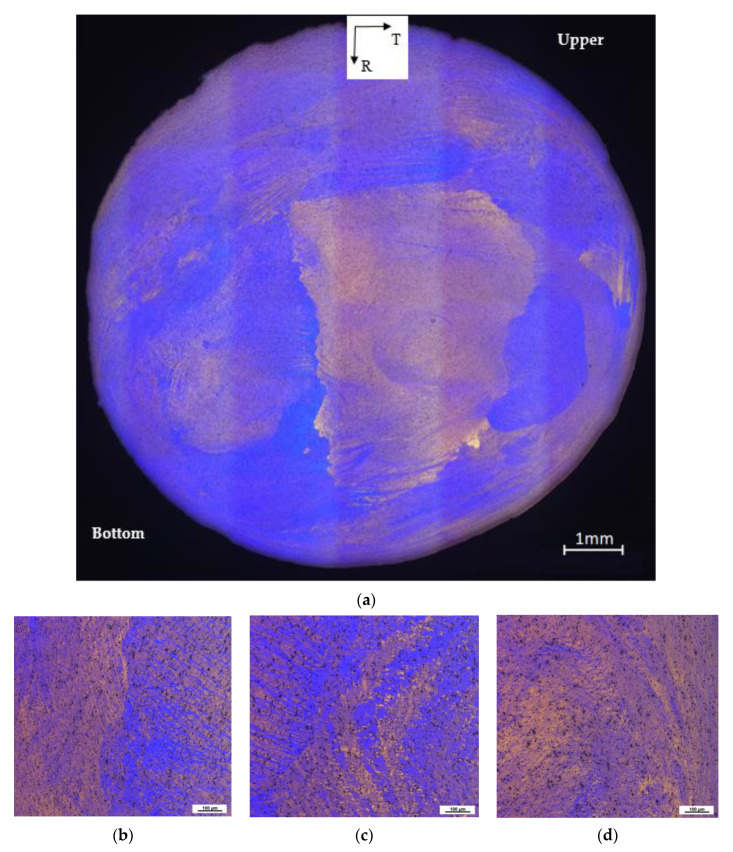
Light micrographs of the ECS material, taken on the TR section: (**a**) overall image of microstructure; (**b**) upper and (**d**) bottom surface region showing a mixture of thin grains in the surface vicinity; and (**c**) massive grains in the center. Barker’s etching was used.

A further investigation of the workpiece was done on its cross-section, where the U surface corresponds to 0 mm in the R direction; see Figure 9a. The cross-section shows the non-homogeneous microstructure, which is a consequence of the asymmetric material flow in the ECAP–Conform tool.

The non-homogeneous microstructure is also visible in Figure 9b–d, where tiny grains are a prevailing feature of the microstructure, especially in the bottom surface region. On the other hand, massive grains were found in the central part of the workpiece (see Figure 9a). The overlapping shear bands of different slip systems in the original grains are visible on the upper surface layer (U); see Figure 9b,d.

**Figure 10:**
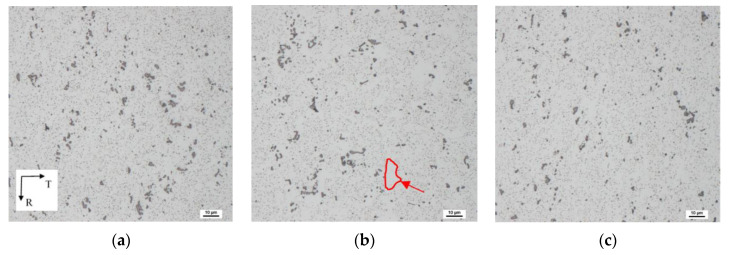
Electron micrographs of the ECS material taken from the TR plane: (**a**) upper and (**c**) bottom surface region; (**b**) central region. Dix–Keller etching was used.

Scanning electron micrographs (Figure 10) of the ECS material taken from the TR section display an array of bigger and smaller precipitates. Precipitate-free zones (marked by the red arrow) are visible in Figure 10b.

**Figure 11:**
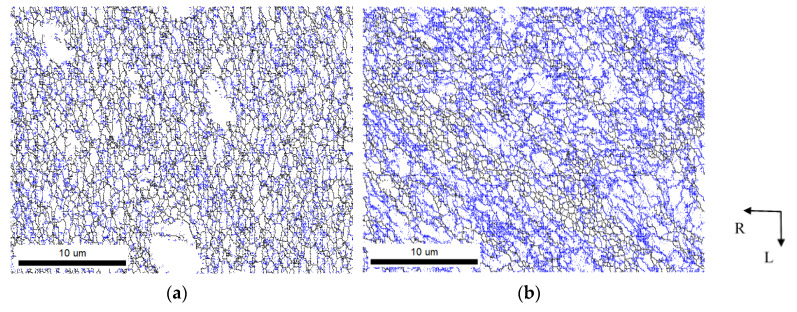

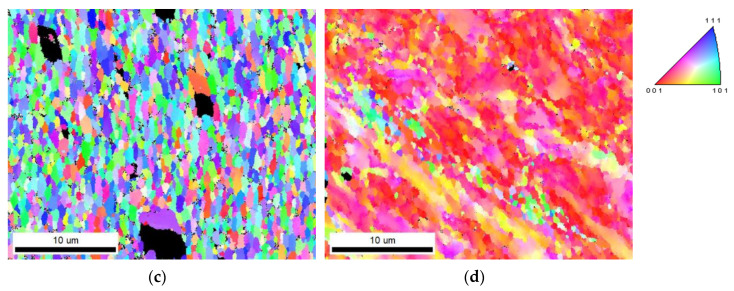
IPF color maps of ECS material showing different crystallographic orientations of grains, indicated based on stereographic triangle projection (LR plane); (**a**) LAGBs (blue) and HAGBs (black) displayed in the bottom surface section; (**b**) LAGBs (blue) and HAGBs (black) displayed in the central section; (**c**) IPF color map displaying grains in the bottom surface region; (**d**) IPF color map displaying grains in the central region.

A deeper investigation of the ECS material was conducted in the section corresponding to the LR plane by means of EBSD analysis, which was taken from the scanned area of 32 × 25 µm^2^. Inverse pole figures (IPF) taken from the bottom surface and central region are reported in Figure 11. The different colors of both pictures indicate different textures existing in the surface and central part of the ECAP–Conformed wire. IPF color mapping shows the formation of low-angle grain boundaries (LAGB) with the disorientation angle of 2° ≤ θ ≤ 15° and high-angle grain boundaries (HAGB) (θ > 15°) in both layers close to the bottom surface, Figure 11a, and to the center, Figure 11b. The mixture of LAGBs (indicated with blue color) and HAGBs (indicated with black color) contains, close to the bottom surface, more HAGBs, in contrast to the central region, where the vast majority of the LAGBs were observed. This indicates a large plastic deformation occurring during the EC process. The HAGBs are concentrated in shear bands, where the smallest grains can also be observed. In the wire central region, the microstructure is uneven, consisting of large and small grains inclined by 45° to the extrusion direction (L). Black places in the IPFs in Figure 11c,d are constituent phase particles. The bimodal distribution of the particle sizes is obvious.

**Figure 12:**
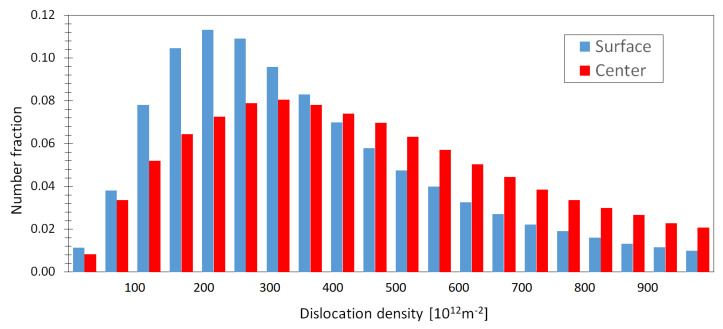
Density of geometrically necessary dislocations estimated in the bottom surface and the central section.

The density of geometrically necessary dislocations (GND) has been estimated using EBSD analysis. Measurement of the dislocation density was done based on the local average grain boundary misorientations shown in Figure 11. A low density of GNDs was found in the bottom surface region (Figure 11c), while in the central part of the wire the dislocation density was higher, as is shown in Figure 11d. The histogram of the density of geometrically necessary dislocations estimated for the bottom surface and the central section is illustrated in Figure 12. This finding is in agreement with the studies of Salehi and coworkers [28]. The average GND density of 368 × 10^12^ m^−2^ and 450 × 10^12^ m^−2^ was measured in the surface and central area of a wire.

**Figure 13:**
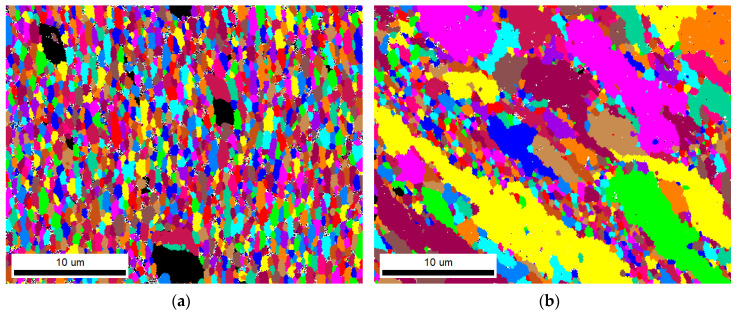
The unique grain color maps of the material after the EC process (LR plane): (**a**) bottom surface region; (**b**) central region.

The different grain sizes existing in both wire regions are clearly visible in Figure 13. The unique grain color maps of the bottom and the central region are presented in Figure 13a,b.

**Figure 14:**
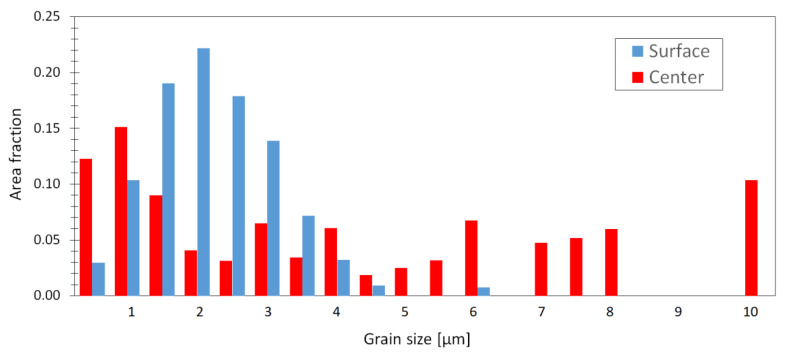
Grain size distributions in the ECS material measured in the tangential sections (TR plane).

Histograms of area fractions of the grain distribution in Figure 14 were obtained based on the unique grain color maps illustrated in Figure 13. Histograms presented are related to the bottom surface (blue) and central (red) section of the wire. This distribution is related to the grain reinforcement in the extrusion direction (L), which was observed in simulation Figure 2. As can be seen in Figure 14, the surface section has a grain size fraction between ~0.2–6 µm, tracking a Gauss distribution. In this region, the average grain size has been found to be 2.0 ± 1.10 µm. On the other hand, the grain size fraction in the central region is very diverse in the range from 0.2 to 10 µm. The grain size distribution in the central region seems to be nearly bimodal, with a significant fraction of ultrafine grains and grains exhibiting a size of several µ; the average grain size was evaluated to be 5.1 ± 2.94 µm.

### 3.2. Micro-Hardness Evaluation

**Figure 15:**
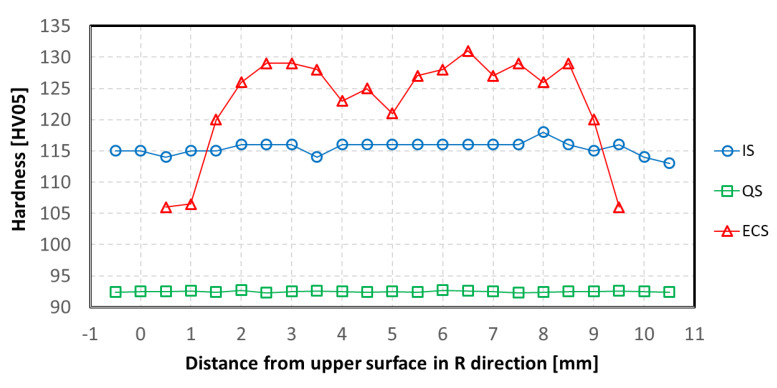
Micro-hardness profile along the cross sections of IS, QS and ECS materials.

Microhardness of the IS and QS materials was measured across the whole wire diameter of 12 mm (see Figure 15). The distance from the upper wire edge (U) corresponds to the distance in the R direction presented in the cutting scheme in Figure 4. The measured values show nearly constant microhardness along the whole wire diameter for both IS and QS states. Lower microhardness values estimated for QS material are a consequence of the HT performed on the IS state. On other hand, a small decrease in the surface region can be seen in the case of the IS state, which corresponds to the metallographic investigation of such extruded material; see Figure 5. Microhardness values estimated for the ECS material show considerable scattering of measurements. Lower microhardness was measured in the edge regions of the sample, near free surface, then the values increased from both sides towards to the sample center. A small microhardness decrease can be seen directly in the wire center, i.e., in the place where bigger grains were detected.

Micro-hardness results for all mentioned states are summarized in Table A1 in the Appendix A for the range of 10 mm.

### 3.3. Tensile Tests

Tensile tests were performed on miniaturized samples in the IS and QS state and for comparison also on samples with a standard geometry prepared from both materials. Two miniaturized flat samples were taken from each material in the extrusion direction (L). IS_L1 and QS_L1 MTT samples were cut out from each wire close to the surface in the edge section and IS_L4 and QS_L4 MTT samples represent the central region of the wire; see schema in Figure 4.

**Figure 16:**
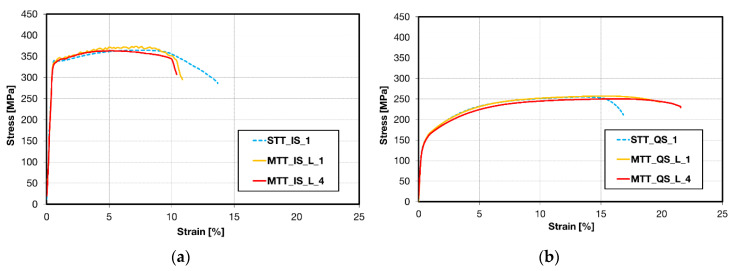
Engineering stress–strain curves obtained for (**a**) IS state and (**b**) QS state.

Engineering stress–strain (S-S) curves obtained for both materials are reported in Figure 16a,b. Only negligible differences in the deformation stresses were observed between standard and miniaturized samples. Values of elongation to fracture vary between the standard and miniaturized tests, but no systematic deviations were observed. Tabled mechanical characteristics, i.e., Young’s modulus (E), yield stress (YS) and ultimate tensile strength (UTS) estimated for both materials, are depicted in Table A2 and Table A3 in the Appendix A. Substantial softening in the QS material compared with the IS state can be seen from Figure 16 and Table A2 and Table A3, as a consequence of the heat treatment.

**Figure 17:**
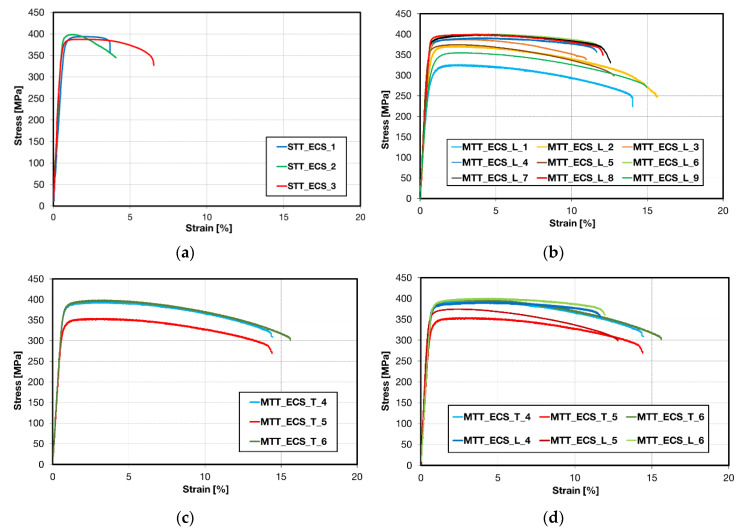
Engineering S-S curves of the ECS material: (**a**) S-S curves performed on standard test samples consisting of three repetitions; (**b**) S-S curves performed on miniaturized samples from the upper edge (MTT_ECS_L1) to the bottom edge (MTT_ECS_L9); (**c**) S-S curves performed on samples in the tangential direction; and (**d**) a comparison of S-S curves obtained using MTT samples in the extrusion (L) and the tangential (T) direction are presented.

Engineering stress–strain (S-S) curves obtained for ECAP–Conformed (ECS) material are shown in Figure 17. Tests were performed on both miniaturized and standard samples. Miniaturized samples were taken from the ECS material in the extrusion direction (L) and perpendicular to this direction (T). E modulus and characteristic stresses obtained in tensile tests for both types of samples and directions are summarized in Table A4 in the Appendix A. Engineering S-S curves of three repetitions of standard samples are presented in Figure 17a. The sequence of engineering S-S curves of miniaturized samples performed in the extrusion direction (L) and in the transversal (T) direction is depicted in Figure 17b,c. The comparison of engineering S-S curves for both directions is shown in Figure 17d.

Significant differences between curves obtained on samples cut from various wire parts reflect various microstructures of samples.

**Figure 18:**
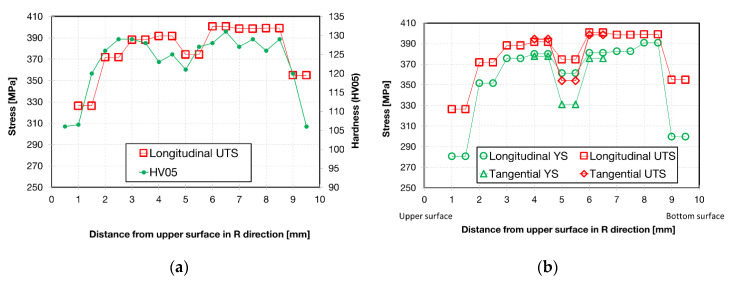
(**a**) UTS and HV05 profile over the wire cross-section; (**b**) YS and UTS profiles measured in longitudinal and tangential directions of the ECS sample.

As it was noted, after the ECAP–Conform process, the micro-hardness profile shows the highest value close to the center of the wire, with a small local minimum directly in the center, as reported in Figure 15. The hardness values increase with increasing distance from the wire edge, reaching their highest hardness values in points 3 or 6 mm from the upper edge. The minimum of hardness was found in the peripheral parts of the wire. In this case, only the hardness measurement may be used for verification of the mechanical properties obtained by the MTT technique. A direct comparison of both results is made in Figure 18a, where the UTS profile exhibits the same shape as the hardness one. The highest strength values were obtained close to the bottom (B) surface of the workpiece, where the highest strain distribution appeared. On the other hand, the tangential direction (T) showed a significant decrease in YS and UTS in the wire center (see Figure 18b) and marginal differences outside of this region.

Table A1, Table A2, Table A3 and Table A4 in the Appendix A summarize results obtained for hardness, YS, UTS and E. The results for Young’s modulus E presented in the Appendix A show a higher scattering of test results, leading to lower values in the case of MTT technique. This can be caused by synchronization of the testing machine and the DIC system. The slight difference in time synchronization of both systems can cause high variance in the measured values. On the other hand, local measurements of mechanical characteristics correspond very well with the microstructure observations.

## 4. Discussion

The ECAP–Conform process may provide severe plastic deformation so as to continuously provide ultrafine-grained materials [7]. The presented results have shown that even one pass through the ECAP–Conform tool is sufficient for substantial improvement of mechanical properties and formation of a refined microstructure. Xu et al. have shown that the main improvement of mechanical properties occurred in the first pass [7]. Th next passes enhanced the yield and ultimate tensile strength only by several percent. On the other hand, the inhomogeneous microstructure after the first pass became more uniform. Material which had undergone one pass in the ECAP–Conform tool exhibited differences between the microstructure observed in the central and peripheral parts on the section perpendicular to the extrusion direction. The grain structure in the peripheral parts exhibited finer grains compared with the central region. The mean grain size measured directly in the edge area of the cross section was lower than the grain size in an annulus between the edge and the cross-section center. The color map analysis revealed that massive grains in the wire center are surrounded by the belts of very small grains. Elongated grains are the characteristic microstructure feature observed in the section parallel to the extrusion direction. The grains’ thickness is larger in the wire center. According to the literature, elongated grains were observed even after four passes in the ECAP–Conform tool [7,17,29]. Note that elongated grain structure is different from the equiaxed grains generally reached in conventional ECAP. It is important, therefore, to perform mechanical testing in order to determine whether there is any plastic anisotropy associated with the testing of samples.

Strain accumulated in the conformed workpiece exhibits several components: bending over the rotating die, decrease in the workpiece cross-section and shear strain realized in the ECAP tool, and strain applied by the frictional forces moving the rod into the tool. FEM confirmed the inhomogeneity of the strain in the conformed wire (see Figure 2). The highest strain values were found in the bottom part and the lowest in the upper part of the wire. The strain difference between both wire edges is very large (see Figure 2). In deformation processing, the mechanical work is mainly dissipated into heat. Therefore, it is reasonable to assume that the bottom part of the wire could have, during deformation processing, a higher temperature; thus, dynamic recovery may locally change the microstructure. Meanwhile, both low strain in the upper part and high strain in the bottom part could very probably cause different mechanical properties in samples from the wire peripheral regions with small grains, where a small number of LAGBs (see Figure 11a) and less GND density are observed. Therefore, these regions have a lower hardness; see Figure 15.

Typical features of the central parts of conformed samples are a mixture of small recrystallized grains and coarser grains having a high density of LAGBs (see Figure 11b). According to Chen and Yu [30], these LAGBs may serve as nucleation sites for new dislocation or as weak barriers that enable selective transmission of dislocations and so contribute to material plasticity.

**Figure 19:**
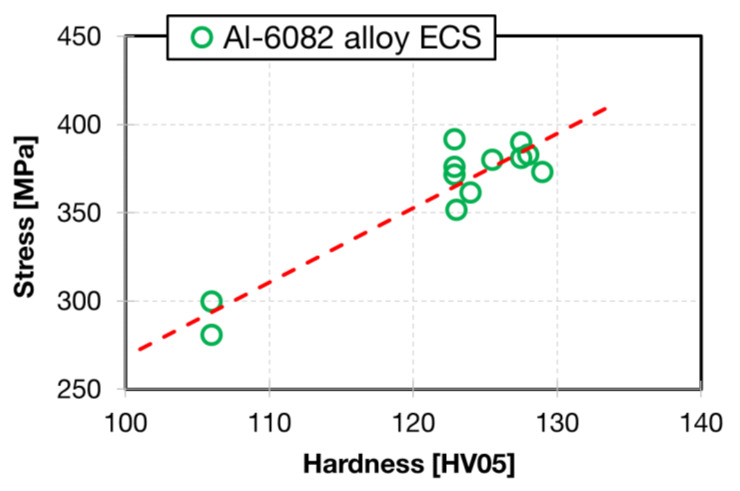
Relationship between hardness HV05 and TYS.

Comparison of measured microhardness values with TYS values estimated for miniaturized and standard tensile tests allowed us to estimate the proportionality parameter in the Tabor relationship [31]:

(1) TYS=C·HV05,

where C is a material parameter which may be estimated experimentally. TYS values plotted against hardness are shown in Figure 19. It is possible to see relatively good correlation between both quantities. The knowledge of C ~ 4 enables a cursory validation of mechanical properties from the hardness measurements.

EBSD analysis has shown the formation of dislocations to be geometrically necessary. This is due to heterogeneous deformation of alloys containing undeformable particles of the second phase [28]. These dislocations are created in order to accommodate large strain gradients and thus allow the two phases to deform in a compatible way. The density of GND is, according to Ashby, calculated as follows [32]:

(2) $$\rho_{G}=\;\;\frac{f\;\;8\varepsilon_{p}}{bt}$$

where *f* is volume fraction of particles, *t* their diameter, b is the Burgers vector of newly created dislocations and ε_p_ is plastic deformation. According to Equation (2), the density of GND depends on the rate of plastic strain. In bigger grains situated in the cross-section center, GNDs are generated and contribute to the alloy strengthening. Calculated dislocation density distributions (see Figure 12) exhibit an increase in GND densities toward the ranges of 10^11^–10^13^ m^−2^. Compared peripheral and central parts’ clear inhomogeneity in the distribution of the GNDs is obvious. This feature is reflected by the hardness measurements and mechanical properties.

Improved mechanical properties may be described by three factors:

- Hall–Petch strengthening due to grain refinement;
- Increased density of statistical dislocations and geometrically necessary dislocations;
- Orowan strengthening.

The present results demonstrate that the MTT technique provides very good material data for material modeling and is fully comparable to conventional specimen test techniques in modeling tensile properties until UTS. In addition, they may be used for evaluation of homogeneity and anisotropy of the ECAP–Conform processed materials that is in high demand these days.

## 5. Conclusions

An ECAP–Conform process was applied on the EN AW 6082 T6 (AlSi1MgMn) aluminum alloy. The microstructural evolution and mechanical properties were characterized. The main results are summarized as follows:

- Even one pass through the ECAP–Conform tool can substantially refine the grain structure.
- This refinement is inhomogeneous; the conformed wire contains bigger grains situated in the center, surrounded by smaller ones.
- Local estimation of mechanical properties showed significant differences between central and peripheral parts of the conformed wire.
- These differences are very probably created by the dissimilar strain in the upper and bottom wire parts.
- Tests performed on transversal samples revealed negligible plastic anisotropy.
- The main strengthening mechanisms are grain size refinement and generation of geometrically necessary dislocations.
- Miniaturized tensile tests are an effective tool for the local estimation of mechanical properties.

## Figures and Tables

**Table 1:** Chemical composition of the alloy.

Element	Si	Fe	Cu	Mn	Mg	Al
wt%	0.91	0.43	0.05	0.85	1.15	bal.

## Appendix A. Tests Results

**Table A1:** Micro-hardness results for IS, QS and ECS states over their cross sections.

	Distance in Radial (R) Direction (mm)																		
	0.5	1.0	1.5	2.0	2.5	3.0	3.5	4.0	4.5	5.0	5.5	6.0	6.5	7.0	7.5	8.0	8.5	9.0	9.5
	**Vickers hardness, HV05**																		
**IS**	114	115	115	116	116	116	114	116	116	116	116	116	116	116	118	116	115	116	116
**QS**	92.5	92.6	92.4	92.7	92.3	92.5	92.6	92.5	92.4	92.5	92.4	92.7	92.6	92.5	92.3	92.4	92.5	92.5	92.6
**ECS**	106	106	120	126	129	129	128	123	125	121	127	128	131	127	129	126	129	120	106

**Table A2:** Tensile test results for IS material.

Sample	E, GPa	YS, MPa	UTS, MPa
STT_IS_1	71.5	339.1	364.5
MTT_IS_L_1	71.6	333.6	370.0
MTT_IS_L_4	70.4	334.5	363.1

**Table A3:** Tensile tests results for QS material.

Sample	E, GPa	YS, MPa	UTS, MPa
STT QS 1	66.7	140.7	254.7
MTT_QS_L_1	68.2	144.3	257.0
MTT_QS_L_4	65.2	147.6	250.3

**Table A4:** Tensile test results for ECS material.

Specimen	E, GPa	YS, MPa	UTS, MPa
STT_ECS_1	65.1	371.6	393.6
STT_ECS_2	71.2	391.5	398.4
STT_ECS_3	73.8	375.8	387.4
MTT_ECS_L_1	58.7	280.5	326.2
MTT_ECS_L_2	56.7	351.6	371.7
MTT_ECS_L_3	61.6	372.9	386.9
MTT_ECS_L_4	60.8	380.2	391.8
MTT_ECS_L_5	69.6	361.9	374.5
MTT_ECS_L_6	61.9	380.6	400.0
MTT_ECS_L_7	62.1	382.0	398.8
MTT_ECS_L_8	62.4	389.4	398.9
MTT_ECS_L_9	62.8	299.7	354.2
MTT_ECS_T_4	58.8	377.7	394.4
MTT_ECS_T_5	57.7	331.1	354.0
MTT_ECS_T_6	60.7	375.7	398.5
